# *N*-(4-Chloro­benzyl­idene)-3,4-dimethyl­isoxazol-5-amine

**DOI:** 10.1107/S1600536810029284

**Published:** 2010-07-31

**Authors:** Abdullah M. Asiri, Salman A. Khan, M. Nawaz Tahir

**Affiliations:** a The Center of Excellence for Advanced Materials Research, King Abdul Aziz University, Jeddah 21589, PO Box 80203, Saudi Arabia; b Department of Chemistry, Faculty of Science, King Abdul Aziz University, Jeddah 21589, PO Box 80203, Saudi Arabia; c Department of Physics, University of Sargodha, Sargodha, Pakistan

## Abstract

The mol­ecule of the title compound, C_12_H_11_ClN_2_O, has *E* configuration at the azomethine double bond and is virtually planar with a dihedral angle of 1.25 (13)° between the benzene and isoxazole rings. C—H⋯π inter­actions stabilize the crystal structure.

## Related literature

For related structures, see: Asiri *et al.* (2010**a* ▶,b* ▶); Fun *et al.* (2010**a* ▶,b* ▶); Shad *et al.* (2008 ▶); Tahir *et al.* (2008 ▶). For graph-set notation, see: Bernstein *et al.* (1995 ▶).
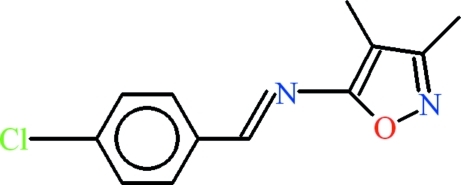


## Experimental

### 

#### Crystal data

C_12_H_11_ClN_2_O
*M* *_r_* = 234.68
Monoclinic, 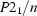

*a* = 5.0877 (2) Å
*b* = 24.5197 (9) Å
*c* = 9.4673 (4) Å
β = 94.871 (2)°
*V* = 1176.77 (8) Å^3^
*Z* = 4
Mo *K*α radiation
μ = 0.30 mm^−1^
*T* = 296 K
0.30 × 0.16 × 0.14 mm

#### Data collection

Bruker Kappa APEXII CCD diffractometer
Absorption correction: multi-scan (*SADABS*; Bruker, 2005 ▶) *T*_min_ = 0.868, *T*_max_ = 0.965
9016 measured reflections
2112 independent reflections
1539 reflections with *I* > 2σ(*I*)
*R*_int_ = 0.030

#### Refinement

*R*[*F* ^2^ > 2σ(*F* ^2^)] = 0.044
*wR*(*F* ^2^) = 0.120
*S* = 1.07
2112 reflections
147 parameters
H-atom parameters constrained
Δρ_max_ = 0.15 e Å^−3^
Δρ_min_ = −0.14 e Å^−3^

### 

Data collection: *APEX2* (Bruker, 2009 ▶); cell refinement: *SAINT* (Bruker, 2009 ▶); data reduction: *SAINT*; program(s) used to solve structure: *SHELXS97* (Sheldrick, 2008 ▶); program(s) used to refine structure: *SHELXL97* (Sheldrick, 2008 ▶); molecular graphics: *ORTEP-3 for Windows* (Farrugia, 1997 ▶) and *PLATON* (Spek, 2009 ▶); software used to prepare material for publication: *WinGX* (Farrugia, 1999 ▶) and *PLATON*.

## Supplementary Material

Crystal structure: contains datablocks text, I. DOI: 10.1107/S1600536810029284/gk2296sup1.cif

Structure factors: contains datablocks I. DOI: 10.1107/S1600536810029284/gk2296Isup2.hkl

Additional supplementary materials:  crystallographic information; 3D view; checkCIF report

## Figures and Tables

**Table 1:** Hydrogen-bond geometry (Å, °) *Cg*1 is the centroid of the O1/N2/C10/C9/C8 ring.

*D*—H⋯*A*	*D*—H	H⋯*A*	*D*⋯*A*	*D*—H⋯*A*
C11—H11*C*⋯*Cg*1^i^	0.96	2.91	3.644 (2)	134

Symmetry code: (i) 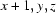.

**Fig. 1.**
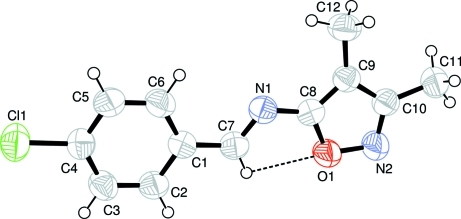
View of the title compound with the atom numbering scheme. The displacement ellipsoids are drawn at the 50% probability level. H-atoms are shown as small spheres of arbitrary radii. The dotted line indicates the intramolecular hydrogen H-bond.

## supplementary crystallographic information

### Comment

The title compound  (Fig. 1) has been prepared in continuation of our work on the synthesis of Schiff bases of 3,4-dimethyisoxazol-5-amine. We have recently reported the crystal structure of N-(4-bromobenzylidene)-3,4-dimethylisoxazol-5-amine (Asiri *et al.*, 2010*a*), which is isostructural with the title compound.

The crystal structures of 4-chloro-2- [(*E*)-({4-[*N*-(3,4-dimethylisoxazol-5-yl)sulfamoyl]phenyl}iminio) methyl]phenolate (Shad *et al.*, 2008), 4-bromo-2-((*E*)-{4-[(3,4-dimethylisoxazol-5-yl)sulfamoyl]phenyl} iminiomethyl)phenolate (Tahir *et al.*, 2008), 2-[(*E*)-(3,4-dimethylisoxazol-5-yl)iminomethyl]phenol (Fun *et al.*, 2010*a*), 1-[(*E*)-(3,4-dimethylisoxazol-5-yl)iminomethyl]-2-naphthol (Fun *et al.*, 2010*b*) and *N*-[4-(dimethylamino)benzylidene]-3,4-dimethylisoxazol-5-amine (Asiri *et al.*, 2010*b*) have also been published previously, which contain the 5-amino-3,4-dimethylisoxazole moiety.

In the title compound, the 4-chlorobenzylidene moiety A (C1—C7/CL1) and 5-amino-3,4-dimethylisoxazole moiety B (N1/C8—C12/N2/O1) are planar with r. m. s. deviation of 0.0042 and 0.0076 Å, respectively. The dihedral angle between A/B is 1.10 (11)°. R. m. s. deviation from the plane of all non-hydrogen atoms in the molecule is 0.0200 Å, with the largest deviation of the CL1 atom [0.0534 (11) Å]. Weak intramolecular H-bonding of C—H···O type (Table 1, Fig. 1) exists and complete an S(5) ring motif (Bernstein *et al.*, 1995). There exists no π···π interaction. The C—H···π interaction (Table 1) play an important role in stabilizing the molecules.

### Experimental

A mixture of 4-chlorobenzaldehyde (0.30 g, 2.2 mmol) and 5-amino-3,4-dimethylisoxazole (0.24 g, 2.2 mmol) in ethanol (15 ml) was refluxed for 5 h with stirring to give a light brown precipitate. This material was filtered off and washed with ethanol to give the pure Schiff base (m.p. 397 K; yield: 78.5%)

^1^*H*-NMR (CDCl_3_) δ: 9.97 (s, 1H, CH_olefinic_), 7.79 (d, H3, CH_aromatic_ J = 5.4 Hz), 7.75 (dd, H4, CH_aromatic_, J = 8.4 Hz), 7.69 (dd, H5, CH_aromatic_ J = 8.4 Hz), 7.61 (d, H6 CH_aromatic_, J = 4.8 Hz), 2.25 (s, N—CH_3_), 1.76 (s,-CH_3_).

### Refinement

The H-atoms were positioned geometrically (C–H = 0.93–0.96 Å) and refined as riding with *U*_iso_(H) = x*U*_eq_(C), where x = 1.5 for methyl and x = 1.2 for other H-atoms.

### Crystal data

**Table:**

C_12_H_11_ClN_2_O	*F*(000) = 488
*M**_r_* = 234.68	*D*_x_ = 1.325 Mg m^−^^3^
Monoclinic, *P*2_1_/*n*	Mo *K*α radiation, λ = 0.71073 Å
Hall symbol: -P 2yn	Cell parameters from 1539 reflections
*a* = 5.0877 (2) Å	θ = 2.3–25.3°
*b* = 24.5197 (9) Å	µ = 0.30 mm^−^^1^
*c* = 9.4673 (4) Å	*T* = 296 K
β = 94.871 (2)°	Needle, light brown
*V* = 1176.77 (8) Å^3^	0.30 × 0.16 × 0.14 mm
*Z* = 4	

### Data collection

**Table:**

Bruker KAPPA APEXII CCD diffractometer	2112 independent reflections
Radiation source: fine-focus sealed tube	1539 reflections with *I* > 2σ(*I*)
graphite	*R*_int_ = 0.030
Detector resolution: 8.10 pixels mm^-1^	θ_max_ = 25.3°, θ_min_ = 2.3°
ω scans	*h* = −6→6
Absorption correction: multi-scan (*SADABS*; Bruker, 2005)	*k* = −29→29
*T*_min_ = 0.868, *T*_max_ = 0.965	*l* = −11→11
9016 measured reflections	

### Refinement

**Table:**

Refinement on *F*^2^	Primary atom site location: structure-invariant direct methods
Least-squares matrix: full	Secondary atom site location: difference Fourier map
*R*[*F*^2^ > 2σ(*F*^2^)] = 0.044	Hydrogen site location: inferred from neighbouring sites
*wR*(*F*^2^) = 0.120	H-atom parameters constrained
*S* = 1.07	*w* = 1/[σ^2^(*F*_o_^2^) + (0.0521*P*)^2^ + 0.2476*P*] where *P* = (*F*_o_^2^ + 2*F*_c_^2^)/3
2112 reflections	(Δ/σ)_max_ < 0.001
147 parameters	Δρ_max_ = 0.14 e Å^−^^3^
0 restraints	Δρ_min_ = −0.14 e Å^−^^3^

### Special details

**Table:**

Geometry. Bond distances, angles *etc*. have been calculated using the rounded fractional coordinates. All su's are estimated from the variances of the (full) variance-covariance matrix. The cell e.s.d.'s are taken into account in the estimation of distances, angles and torsion angles
Refinement. Refinement of *F*^2^ against ALL reflections. The weighted *R*-factor *wR* and goodness of fit *S* are based on *F*^2^, conventional *R*-factors *R* are based on *F*, with *F* set to zero for negative *F*^2^. The threshold expression of *F*^2^ > σ(*F*^2^) is used only for calculating *R*-factors(gt) *etc*. and is not relevant to the choice of reflections for refinement. *R*-factors based on *F*^2^ are statistically about twice as large as those based on *F*, and *R*- factors based on ALL data will be even larger.

### Fractional atomic coordinates and isotropic or equivalent isotropic displacement parameters (Å^2^)

**Table:**

	*x*	*y*	*z*	*U*_iso_*/*U*_eq_	
Cl1	−0.59118 (13)	0.19483 (3)	−0.44471 (7)	0.0894 (3)	
O1	0.5343 (3)	0.03641 (6)	0.15781 (16)	0.0736 (6)	
N1	0.3653 (3)	0.11804 (7)	0.05045 (17)	0.0608 (6)	
N2	0.7399 (4)	0.02071 (8)	0.2600 (2)	0.0763 (7)	
C1	−0.0022 (4)	0.11701 (8)	−0.1255 (2)	0.0576 (7)	
C2	−0.1918 (4)	0.08576 (9)	−0.2009 (2)	0.0676 (8)	
C3	−0.3750 (4)	0.10921 (9)	−0.2987 (2)	0.0701 (8)	
C4	−0.3674 (4)	0.16448 (9)	−0.3200 (2)	0.0630 (8)	
C5	−0.1824 (5)	0.19639 (9)	−0.2446 (3)	0.0775 (9)	
C6	−0.0019 (4)	0.17261 (10)	−0.1484 (3)	0.0724 (8)	
C7	0.1915 (4)	0.09088 (9)	−0.0251 (2)	0.0622 (7)	
C8	0.5459 (4)	0.09160 (9)	0.1431 (2)	0.0576 (7)	
C9	0.7462 (4)	0.11227 (8)	0.2291 (2)	0.0575 (7)	
C10	0.8603 (4)	0.06588 (9)	0.2988 (2)	0.0617 (7)	
C11	1.0916 (4)	0.06456 (11)	0.4077 (2)	0.0785 (9)	
C12	0.8318 (4)	0.16992 (9)	0.2438 (3)	0.0778 (9)	
H2	−0.19609	0.04832	−0.18554	0.0811*	
H3	−0.50149	0.08784	−0.34921	0.0842*	
H5	−0.18016	0.23391	−0.25895	0.0930*	
H6	0.12298	0.19425	−0.09757	0.0869*	
H7	0.18824	0.05313	−0.01578	0.0746*	
H11A	1.15318	0.02772	0.42002	0.1177*	
H11B	1.03940	0.07821	0.49613	0.1177*	
H11C	1.23056	0.08692	0.37665	0.1177*	
H12A	0.72003	0.19230	0.18085	0.1168*	
H12B	1.01097	0.17312	0.22023	0.1168*	
H12C	0.81971	0.18168	0.33972	0.1168*	

### Atomic displacement parameters (Å^2^)

**Table:**

	*U*^11^	*U*^22^	*U*^33^	*U*^12^	*U*^13^	*U*^23^
Cl1	0.0947 (5)	0.0856 (5)	0.0842 (4)	0.0155 (3)	−0.0142 (3)	0.0044 (3)
O1	0.0787 (10)	0.0567 (9)	0.0821 (10)	−0.0045 (7)	−0.0123 (8)	−0.0012 (7)
N1	0.0579 (10)	0.0613 (11)	0.0634 (10)	−0.0002 (8)	0.0067 (8)	−0.0017 (8)
N2	0.0797 (12)	0.0638 (12)	0.0816 (12)	0.0000 (10)	−0.0161 (10)	0.0055 (10)
C1	0.0568 (11)	0.0568 (12)	0.0597 (11)	0.0007 (9)	0.0079 (9)	−0.0057 (9)
C2	0.0741 (14)	0.0517 (12)	0.0761 (14)	−0.0019 (10)	0.0014 (11)	−0.0053 (11)
C3	0.0702 (14)	0.0664 (14)	0.0715 (14)	−0.0028 (11)	−0.0071 (11)	−0.0110 (11)
C4	0.0642 (13)	0.0644 (14)	0.0602 (12)	0.0080 (10)	0.0041 (10)	−0.0028 (10)
C5	0.0836 (16)	0.0537 (13)	0.0932 (17)	−0.0027 (11)	−0.0041 (13)	0.0021 (12)
C6	0.0709 (14)	0.0617 (14)	0.0822 (15)	−0.0064 (11)	−0.0074 (11)	−0.0056 (12)
C7	0.0663 (13)	0.0559 (12)	0.0647 (12)	0.0006 (10)	0.0077 (10)	−0.0048 (10)
C8	0.0589 (12)	0.0548 (12)	0.0598 (11)	0.0013 (9)	0.0099 (9)	−0.0006 (9)
C9	0.0565 (11)	0.0618 (13)	0.0552 (11)	−0.0041 (10)	0.0102 (9)	−0.0011 (10)
C10	0.0633 (12)	0.0647 (13)	0.0575 (11)	−0.0039 (11)	0.0082 (9)	0.0004 (10)
C11	0.0716 (14)	0.0913 (18)	0.0708 (14)	−0.0056 (12)	−0.0044 (11)	0.0059 (12)
C12	0.0793 (15)	0.0613 (14)	0.0921 (16)	−0.0106 (12)	0.0026 (12)	−0.0055 (12)

### Geometric parameters (Å, °)

**Table:**

Cl1—C4	1.737 (2)	C9—C10	1.415 (3)
O1—N2	1.417 (2)	C9—C12	1.482 (3)
O1—C8	1.362 (3)	C10—C11	1.498 (3)
N1—C7	1.277 (3)	C2—H2	0.9300
N1—C8	1.377 (3)	C3—H3	0.9300
N2—C10	1.303 (3)	C5—H5	0.9300
C1—C2	1.382 (3)	C6—H6	0.9300
C1—C6	1.380 (3)	C7—H7	0.9300
C1—C7	1.458 (3)	C11—H11A	0.9600
C2—C3	1.382 (3)	C11—H11B	0.9600
C3—C4	1.371 (3)	C11—H11C	0.9600
C4—C5	1.376 (3)	C12—H12A	0.9600
C5—C6	1.368 (4)	C12—H12B	0.9600
C8—C9	1.348 (3)	C12—H12C	0.9600
			
Cl1···H12C^i^	3.0600	C12···H11C	3.0700
O1···H7	2.3400	H2···H7	2.4300
O1···H2^ii^	2.7200	H2···O1^ii^	2.7200
N1···H6	2.5800	H3···C11^vi^	3.0200
N1···H12A	2.7800	H6···N1	2.5800
N1···H12B^iii^	2.8500	H7···O1	2.3400
C3···C7^iii^	3.570 (3)	H7···H2	2.4300
C7···C3^iv^	3.570 (3)	H11B···C3^vii^	3.0800
C7···C9^iii^	3.482 (3)	H11C···C8^iv^	2.8400
C9···C7^iv^	3.482 (3)	H11C···C12	3.0700
C3···H11B^i^	3.0800	H12A···N1	2.7800
C8···H11C^iii^	2.8400	H12B···N1^iv^	2.8500
C11···H3^v^	3.0200	H12C···Cl1^vii^	3.0600
			
N2—O1—C8	107.69 (15)	C1—C2—H2	119.00
C7—N1—C8	120.28 (18)	C3—C2—H2	119.00
O1—N2—C10	105.29 (17)	C2—C3—H3	121.00
C2—C1—C6	118.45 (19)	C4—C3—H3	121.00
C2—C1—C7	119.77 (18)	C4—C5—H5	120.00
C6—C1—C7	121.78 (19)	C6—C5—H5	120.00
C1—C2—C3	121.1 (2)	C1—C6—H6	119.00
C2—C3—C4	118.94 (19)	C5—C6—H6	119.00
Cl1—C4—C3	119.90 (16)	N1—C7—H7	119.00
Cl1—C4—C5	119.28 (18)	C1—C7—H7	119.00
C3—C4—C5	120.82 (19)	C10—C11—H11A	109.00
C4—C5—C6	119.6 (2)	C10—C11—H11B	109.00
C1—C6—C5	121.1 (2)	C10—C11—H11C	109.00
N1—C7—C1	122.3 (2)	H11A—C11—H11B	109.00
O1—C8—N1	120.02 (18)	H11A—C11—H11C	109.00
O1—C8—C9	110.42 (18)	H11B—C11—H11C	109.00
N1—C8—C9	129.6 (2)	C9—C12—H12A	109.00
C8—C9—C10	103.81 (18)	C9—C12—H12B	109.00
C8—C9—C12	128.04 (19)	C9—C12—H12C	109.00
C10—C9—C12	128.13 (19)	H12A—C12—H12B	109.00
N2—C10—C9	112.79 (18)	H12A—C12—H12C	109.00
N2—C10—C11	119.9 (2)	H12B—C12—H12C	110.00
C9—C10—C11	127.3 (2)		
			
C8—O1—N2—C10	−0.4 (2)	C1—C2—C3—C4	−0.3 (3)
N2—O1—C8—N1	−179.39 (17)	C2—C3—C4—Cl1	178.75 (15)
N2—O1—C8—C9	0.1 (2)	C2—C3—C4—C5	−0.6 (3)
C8—N1—C7—C1	−179.45 (18)	Cl1—C4—C5—C6	−178.59 (19)
C7—N1—C8—O1	−2.1 (3)	C3—C4—C5—C6	0.8 (4)
C7—N1—C8—C9	178.5 (2)	C4—C5—C6—C1	0.0 (4)
O1—N2—C10—C9	0.5 (2)	O1—C8—C9—C10	0.2 (2)
O1—N2—C10—C11	179.91 (17)	O1—C8—C9—C12	178.5 (2)
C6—C1—C2—C3	1.0 (3)	N1—C8—C9—C10	179.6 (2)
C7—C1—C2—C3	−178.80 (18)	N1—C8—C9—C12	−2.1 (4)
C2—C1—C6—C5	−0.8 (3)	C8—C9—C10—N2	−0.4 (2)
C7—C1—C6—C5	179.0 (2)	C8—C9—C10—C11	−179.78 (19)
C2—C1—C7—N1	−177.71 (19)	C12—C9—C10—N2	−178.7 (2)
C6—C1—C7—N1	2.5 (3)	C12—C9—C10—C11	1.9 (3)

Symmetry codes: (i) *x*−1, *y*, *z*−1; (ii) −*x*, −*y*, −*z*; (iii) *x*−1, *y*, *z*; (iv) *x*+1, *y*, *z*; (v) *x*+2, *y*, *z*+1; (vi) *x*−2, *y*, *z*−1; (vii) *x*+1, *y*, *z*+1.

### Hydrogen-bond geometry (Å, °)

**Table:**

Cg1 is the centroid of the O1/N2/C10/C9/C8 ring.

**Table:**

*D*—H···*A*	*D*—H	H···*A*	*D*···*A*	*D*—H···*A*
C7—H7···O1	0.93	2.34	2.704 (3)	103
C11—H11C···Cg1^iv^	0.96	2.91	3.644 (2)	134

Symmetry codes: (iv) *x*+1, *y*, *z*.
